# Intrarenal resistance index for the assessment of acute renal injury in a rat liver transplantation model

**DOI:** 10.1186/1471-2369-14-55

**Published:** 2013-03-02

**Authors:** Hai-Ying Kong, Fen Chen, Yong He, Lin-Jiao Wu, Li-Qing Wang, Sheng-Mei Zhu, Shu-Sen Zheng

**Affiliations:** 1 Department of Anesthesiology, the First Affiliated Hospital, Zhejiang University School of Medicine, Hangzhou, 310003, PR China; 2 Key Lab of combined Multi-organ Transplantation, Ministry of Public Health, the First Affiliated Hospital, Zhejiang University School of Medicine, 79 Qingchun Road, Hangzhou 310003, PR China; 3 State Key Laboratory for Diagnosis and Treatment of Infectious Diseases; Key Laboratory of Infectious Diseases, Zhejiang Province; Department of Infectious Diseases, First Affiliated Hospital, Zhejiang University School of Medicine, Hangzhou, 310003, PR China

**Keywords:** Liver transplantation, Doppler sonography, Renal function, Resistive index, Cys, NGAL

## Abstract

**Background:**

Acute kidney injury (AKI) is a common complication after liver transplantation (LT) and associated with a high mortality. The renal resistive index (RI) is used to assess early renal function impairment in critical care patients. However, limited data are available concerning changes of renal RI and the development of AKI early after reperfusion. We approached to investigate the changes of renal RI and AKI after reperfusion in a rat liver transplantation model.

**Methods:**

Rats were randomly divided into sham group or LT group. Ten rats in each group were used for the hemodynamic study and twenty for Doppler measurements during the procedure. Ten rats were sacrificed 30 min or 2 h after the reperfusion. We harvested kidneys, serum and urine for further analysis of the renal function.

**Results:**

The intrarenal RI increased significantly in the anhepatic stage and decreased significantly after the reperfusion in the LT group compared with sham group (P < 0.05). AKI was seen after the reperfusion in the LT group. No correlation was noted between the RI and renal function parameters 30 min after reperfusion.

**Conclusions:**

The intrarenal RI increased significantly during the anhepatic stage, and decreased significantly early after the reperfusion. Intrarenal RI was unable to assess renal function in a rat liver transplantation model.

## Background

AKI is one of the most common complications after LT, especially in the early postoperative period [1-6]. Although the precise cause of the renal injury after LT remains elusive and is likely multifactor [2,5,7-9], renal vascular tone is one of the factors closely related to the effective renal perfusion and subsequent renal function [10-12]. In many cases, such as hemorrhagic shock, immediate elevations in renal RI have been reported and renal injury may occur as a functional disorder secondary to splanchnic pooling of blood, reduced effective arterial volume and compensatory activation of vasopressor systems leading to increased renal vascular tone [13]. The pathophysiology of human liver transplantation was more complex than hemorrhage shock, and there would be acute changes in intraoperative hemodynamics resulting from suprarenal inferior vena cava (IVC) occlusion, secondary to ischemia-reperfusion injury in the liver in the procedure [3,6], and would induce major changes in renal vascular tone. However, limited data related to renal vascular tone and renal function directly after reperfusion existed during LT [11,12].

The Doppler waveform analysis of the kidneys, semiquantified as the RI, has accumulated for both acute and chronic renal diseases [11,12,14,15]. It has also been thought to indirectly reflect the degree of resistance in the intrarenal vasculature [16] and is gaining growing attention as an important factor predicting the occurrence of AKI in critical care patients [14,17]. The patients with an elevated RI were at greater risk for development of AKI and overt hepatorenal syndrome [14]. Early identification of the renal vascular tone and development of AKI in this special patient population may be beneficial because clinical therapies may be modified to avoid other nephrotoxins and to improve against AKI.

As serum Cystatin C(Cys), a more accurate predictive biomarker of glomerular filtration rate (GFR), and neutrophil gelatinase-associated lipocalin (NGAL), an early predictive biomarker of AKI, have been successfully used in the liver diseases and LT studies [4,6,18-22], we approached to investigate the changes of renal RI values during anhepatic stage and early neohepatic stage as well as the Cys, NGAL after reperfusion in a rat liver transplantation model. This investigation might generate new insights into the response of the kidney to potential harmful factors during the early reperfusion and raise the concern into the diagnostic value of RI measurements alone.

## Methods

### Animals

Male Sprague–Dawley rats, aged 8–10 wks, weighing 200–250 g, purchased from the Animal Resource Center at Zhejiang University School of Medicine, were used as donors and recipients. All the animal research protocols used in this study were approved by the Animal Ethics Review Committees of Zhejiang University and accorded with the principles stated in the Guide for the Care and Use of Laboratory Animals (National Institutes of Health publication, 1985).

### Experimental design and surgical procedure

The rats were randomly divided into two groups: sham operation group (Sham group; n = 50) and orthotopic LT group (LT group; n = 50). Ten recipients in each group were chosen for hemodynamic study; ten for examination of Doppler ultrasound during the anhepatic stage and ten for examination after the reperfusion; and 30 min or 2 h after the reperfusion, ten recipients were sacrificed, and blood and kidney samples were collected for further analysis.

Orthotopic liver syngenic graft transplantations were performed with the two-cuff technique, which was first established by Kamada [23]. The rats were intraperitoneally injected with chloral hydrate anesthesia, 200 mg.kg^-1^ body weight or to effect. During the surgery, the rats were allowed to breathe oxygen on an electric heating pad under a warming light. The graft was stored in cold saline with a target cold ischemic time of 80 min; More than 90% of the rats survived this surgery. To compensate for insensible water loss and fasting period, each animal was given lactated Ringer’s 6 ml/kg per hour using a two-channel infusion pump (Anne; Abbott) during animal preparation and liver section. There was no administration of vasoconstrictors and calcinenrin inhibitor during the operation.

### Hemodynamic study

Ten rats in each group were used for hemodynamic study. After induction of anesthesia, left cervical arteries were cannulated by a catheter for measurement of mean arterial pressure (MAP). The catheter was connected via the pressure transducer (YPJ01 Pressure Transducer, physiological experiment system, Chengdu Instruments, Sichuan, China) to a multichannel data-recording unit (RM6240C, physiological experiment system, Chengdu Instruments) for continuous pressure monitoring and recording. MAP and HR were recorded before removing the liver, at the time of clamping and after the reperfusion. All data were analyzed using the physiological experiment software system (RM6240 physiological experiment system, Chengdu Instruments).

### Doppler measurements

Colour-coded duplex sonography was performed using an Esaote Mylab duplex device with a curved 2–5 MHz transducer following a standardized protocol [24]. Baseline Doppler ultrasound examinations (Esaote Mylab90, XVISION, IOE323, Italy) and calculation of RI were performed on the right kidney. Three consecutive measurements at segmental and arcuate renal arteries on the right kidney were averaged. RI values were calculated automatically by the ultrasound machine using standard methodology (RI = [peak systolic velocity-peak diastolic velocity]/peak systolic velocity). RI assessments were made 10 min, 15 min after clamping of the portal vein and IVC; 5 min, 10 min, 15 min, 20 min, and 30 min after the reperfusion. RI assessments were also made at the above time points in the sham group. All Doppler examinations were done by one examiner (CF).

### Assessment of liver and renal function after reperfusion

Blood samples were collected from the recipients 30 min and 2 h after the reperfusion (10 rats for sampling at each time point) and processed within 2 h after collection. Blood collected in serum separator tubes was allowed to clot for 15–20 min and then centrifuged for 12 min at 1000 g. Serum was collected and subsequently frozen at −20°C until further analysis: 50 μl for the measurement of alanine aminotransferase (ALT), aspartate aminotransferase (AST), concentrations of Na^+^ and serum creatinine activities, reported in units per liter (Hitachi 747 Automatic Analyzer; Boehringer Mannheim GmbH, Mannheim, Germany); and 50 μl for NGAL immunoassay. Quantitative NGAL levels were measured with a sandwich enzymelinked immunosorbent assay (R&D Systems, Minneapolis, Minn., USA) according to the manufacturer’s instructions. Concentrated samples were diluted up to 80-fold with the manufacturer-provided diluent. Standards, samples and controls were run in duplicate, and the resulting chromogen was read at 450 nm with an additional 570-nm wavelength correction (Tecan, San Jose, Calif., USA). NGAL concentrations (ng/ml) were then calculated on the basis of the constructed standard curves on respective enzyme-linked immunosorbent assay plates. Standard curve R^2^ values ranged from 0.9903 to 0.9987. And another 50 μl was used for Cys immunoassay. Quantitative Cys levels were measured with a sandwich enzymelinked immunosorbent assay (R&D Systems, Minneapolis, Minn., USA) according to the manufacturer’s instructions. Urine samples were collected during the reperfusion period, and the volume of urine produced was recorded. Urine concentrations of Na^+^ and creatinine were measured (Vetlab Services) and the FE_Na_ was calculated as [(urine sodium/ plasma sodium)/(urine creatinine/plasma creatinine)] × 100.

### Histology and Quantification of Renal Injury

The kidney sections were stained with hematoxylin–eosin and periodic-acid Schiff. Samples were analyzed for tubular cell necrosis, tubular dilation, intratubular detachment (×20), and evaluated in a blinded manner by a nephrologist. Abnormalities were graded by a semiquantitative score (0 to 4^+^): 0, no abnormalities; 1^+^,changes affecting < 25% of the tubules; 2^+^, 25% to 50%;3^+^, 50% to 75%; 4^+^, >75%.

### Statistical analysis

All data were presented as mean ± standard deviations (SD), and statistical analyses were performed using SAS release 6.12 (SAS Institute, Cary, NC). Data between experimental groups were compared using a 2-tailed unpaired *t* test. Correlation of RI values,Cys and NGAL was tested by linear regression. Significance was defined as P < 0.05.

## Results

### Hemodynamic changed during liver transplantation

In the LT group, MAP decreased slightly while HR increased significantly during the anhepatic stage. After a compensatory increase of MAP and decrease of HR during the initial reperfusion stage, the MAP, HR changed gradually toward the value recorded at baseline after the reperfusion in the LT recipients (Table 1). In the sham group, the MAP and HR are stable during the operation.

**Table 1:** Hemodynamic variables of the two groups during the different phases of liver transplantation

**Parameters**	**BL**	**C5**	**R5**	**R15**	**R30**	**R120**
MAP(mmHg)						
Sham group	80 ± 6.3	79 ± 6	82 ± 6.4	81 ± 6.1	80 ± 6.2	83 ± 6
LT group	83 ± 6.5	54 ± 4.8^*^	64 ± 5.2^*^	66 ± 5.5^*^	68 ± 6.3^*^	76 ± 7.1
HR(bpm)						
Sham group	80 ± 7.5	82 ± 9.3	84 ± 9.0	82 ± 8.1	80 ± 8.7	81 ± 9.5
LT group	81 ± 6.3	114 ± 11.2^*^	98 ± 10.6^*^	94 ± 9.8^*^	90 ± 8.5^*^	93 ± 9.6^*^

Values are expressed as mean ± SD. *: *P* < 0.05, vs. sham group.

Sham: sham operating; LT: liver transplantation. BL: baseline; C_5_: 5 min after portal vein clamping; R_5_, R_15_, R_30_, R_120_: 5 min, 15 min, 30 min, 120 min after reperfusion.

### Renal resistive index and arterial velocity

**Figure 1:**
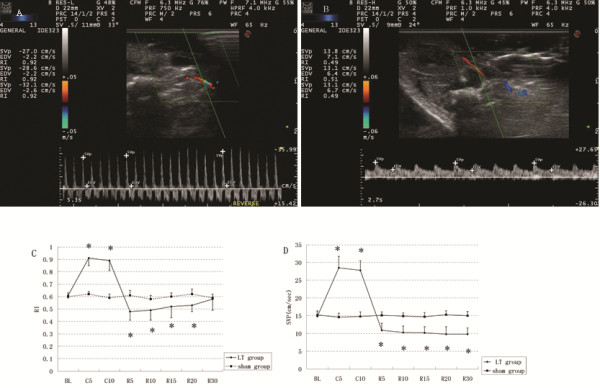
**The right intrarenal RI recorded 10 min after portal vein and IVC clamping (A); The right intrarenal RI recorded 10 min after reperfusion in the recipient in the LT group (B). **Renal RI recorded in the anhepatic stage and early after reperfusion (**C**); arterial velocity recorded in the anhepatic stage and early after reperfusion (**D**). Results are expressed as mean ± SD. *: *P* < 0.05, vs. sham group. BL: baseline; C_5_: 5 min after portal vein clamping, C_10_: 10 min after portal vein and IVC clamping; R_5_, R_10_, R_15_, R_20_, R_30_: 5 min, 10 min, 15 min, 20 min, 30 min after reperfusion; SVP: peak arterial systolic velocity.

Renal resistive index and arterial velocity changed significantly during the procedure (Figure 1). The right intrarenal RI values increased significantly 10 min, 15 min after clamping IVC and portal vein, but decreased significantly 5 min, 10 min, 15 min, 20 min after reperfusion compared with sham group (Figure 1); The median peak arterial velocity increased significantly in the anhepatic stage and decreased significantly after the reperfusion compared with sham group (Figure 1).

### Liver function and Renal function after the reperfusion

The ALT and AST 30 min and 2 h after the reperfusion (Table 2) were significantly increased compared with sham group (*P* < 0.05). We measured plasma creatinine, Cys, NGAL 30 min and 2 h after the reperfusion. The serum levels of the three markers were increased 30 min, 2 h after the reperfusion significantly compared with sham group (Table 2). To better describe the behavior of diuresis and fractional sodium excretion, we also measured FE_Na_ 30 min, 2 h after the reperfusion. The FE_Na_ increased significantly compared with sham group (Table 2). There was no significant correlation between the RI and renal function parameters (sCr, Cys, NGAL) 30 min after the reperfusion (P > 0.05).

**Table 2:** Comparison of liver function and renal function 30 min, 2 h after reperfusion in the two groups

**Parameters**	**30 min**	**2 h**
ALT(U/L)		
Sham group	25.2 ± 10.1	26.1 ± 9.8
LT group	317.56 ± 106.24^*^	405 ± 108.07^*^
AST(U/L)		
Sham group	45.6 ± 10.4	47.2 ± 9.8
LT group	412 ± 109.12^*^	846.13 ± 213.51^*^
sCr(mg/dL)		
Sham group	7.4 ± 2.3	8.2 ± 2.5
LT group	36.1 ± 13.2^*^	34.7 ± 11.2^*^
NGAL(mg/L)		
Sham group	0.83 ± 0.11	0.91 ± 0.15
LT group	1.53 ± 0.37^*^	1.72 ± 0.39^*^
Cys(mg/L)		
Sham group	0.11 ± 0.03	0.14 ± 0.02
LT group	1.32 ± 0.33^*^	1.18 ± 0.46^*^
FE_Na_(%)		
Sham group	0.28 ± 0.04	0.31 ± 0.05
LT group	1.22 ± 0.14^*^	1.28 ± 0.12^*^

Values are expressed as mean ± SD. *: *P* < 0.05, vs. sham group.

Sham: sham operating; LT: liver transplantation.

### Renal Histology

**Figure 2:**
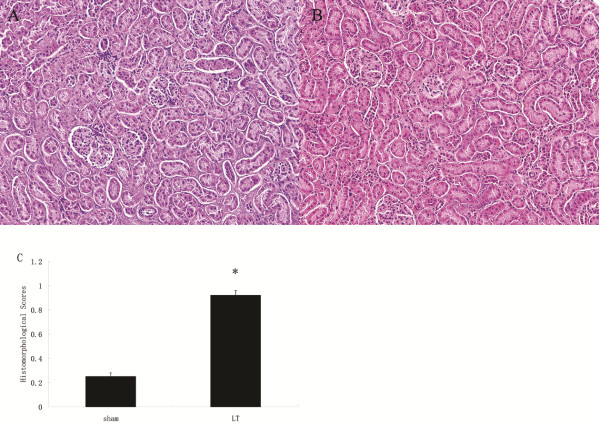
**Histological evaluation of renal tissue obtained 2 h after reperfusion. **A hemotoxylin-eosin stain of kidney sections in the two groups(**A**- sham group, **B**- LT group); (**C**) Semiquantitative scoring of histological injury (n = 10). Data are expressed as the mean ± SD *: *P* < 0.05, vs. sham group. Magnification: 20×.

In the renal sections, we demonstrated multifocal acute tubular injury as evidenced by loss of brush border, flattening and loss of tubular epithelium, hyaline casts, medullary congestion, and hemorrhage. The tubular injury in the LT group was presented while the renal morphology of sham rats was near normal (Figure 2).

## Discussion

LT patients run the risk of developing AKI and subsequent chronic kidney disease, affecting morbidity and mortality [1-3]. Ischemia is the most common cause of AKI; ischemic-induced renal tissue hypoxia is thought to be a major component in the development of AKI in promoting the initial tubular damage [25]. Ensuring the adequate perfusion and oxygenation of the kidney is central in the prevention of AKI [13].

Colour coded duplex sonography is the accepted method to assess kidney perfusion after transplantation [12,17,24,26]. A widely used Doppler parameter is the intrarenal RI. A better knowledge of the changes and diagnostic value of the intrarenal RI during the procedure will assist in designing studies to prevent AKI.

This study provided information on the physiology of renal blood flow during the LT. The most striking findings were the increased renal RI in the anhepatic stage and decreased renal RI early after the reperfusion in a rat liver transplantation model. These results were different from the reports from Platt [26] and Pompili [12], which showed that the increased RI before LT can revert to normal a week after transplantation. RI measured at the different points can partially explain this apparent discrepancy, the RI values were initially measured on the first day after the operation but not immediately after the reperfusion, and the renal vasoconstriction due to the administration of cyclosponine (CSA) after operation can increase renal RI. The administration of CSA was associated with alterations in adrenergic tone and activation of the renin-angiotensin system [27].

The high renal RI values and arterial velocities in the anhepatic stage may be due to the acute changes in intraoperative hemodynamics secondary to the clamping of the vena cava. Clamping of the vena cava can result in the decreased cardiac preload and subsequent pronounced reduction in blood pressure; it also can increase caval and renal vein pressures and subsequently decrease the renal perfusion pressure (mean arterial pressure minus renal vein pressure) [3]. The high RI values suggested that the renal response to acute changes in hemodynamics during the anhepatic stage is similar to that in hemorrhage shock, relative hypovolemia may result in vasoconstriction and increased vascular resistance [13]. Substantial maldistribution of blood flow to the kidney might be present during the anhepatic stage.

In contrast to the findings in the anhepatic stage, the pronounced reduction in RI values early after the reperfusion compared with baseline was a novel finding [12,26]. The factors influencing the RI include HR, arterial stenosis, vein thrombosis and renal edema [12]. In this study, HR 5 min after clamping the portal vein and IVC was increased compared with that recorded 5 min after the reperfusion, thus the effect of the HR on the RI can be neglected. Although the mechanical renal artery stenosis can’t occur as no intervention involving renal artery in LT, an increased number of thrombotic or inflammatory can obstruct capillaries in the corticomedullary junction and influence the vascular resistance [28]. The increased MPO activity in the renal tissue, suggested the leukocyte activation and infiltration occurred in renal tissue during LT [29]. In addition, the elevated NGAL after the reperfusion suggested that acute renal tubular injury occurred after the reperfusion. Furthermore, renal edema can also develop due to secondary to ischemia-reperfusion injury and venous ischemia [28]. Hence, the renal RI was expected to elevate after the reperfusion due to the changes in the renal parenchyma.

Thus, the declined renal RI values early after the reperfusion might be ascribed to the reduction of tone within renal arterioles, resulting from the imbalance between vasoconstrictive and vasodilative factors. During LT, on the reperfusion of the implanted liver, the graft itself can release substances such as proinflammatory cytokines [3,30,31] and nitric oxide [32], which can lead to the imbalance between vasoconstrictive and vasodilative factors and subsequently disturbance the adapt capacity of vascular resistance and renal vascular dilates [31,33]. Furthermore, this reduction of tone within renal might overwhelm the elevated renal RI values related to leukocyte activation and infiltration, renal tubular injury and renal edema.

Usually, reduced renal vascular tone would also imply increased renal blood flow and improve renal function [12,17]. However, the impaired renal function including decreased GFR and renal tubule injury was presented immediately after the reperfusion in this rat liver transplantation model. The increased Cys suggested that the GFR might decrease significantly after the reperfusion. We also showed marked renal tubule injury including increased NGAL and FE_Na_. FE_Na_ has been used in the diagnosis of acute renal failure (ARF) to distinguish between the two main causes of ARF, prerenal state and acute tubular necrosis (ATN) [34,35]. The activation of neurohumoral systems (angiotensin, endothelin, and catecholamines) induced by the hemodynamic changes in portal hypertention-induced arterial vascular underfilling in liver cirrhosis can increase sodium reabsorption [36], and result in FE_Na_ <1%. Our data showed that FE_Na_ increased after the reperfusion and levels > 1%, indicating the presence of ATN after the reperfusion. The histologic evidence of tubule injury seen in the rats after the reperfusion also suggested that rats suffered AKI after the reperfusion. Similar findings were also reported before [29,37]. Postreperfusion syndrome [8] and surgical technique [6] have been pointed as risk factors related to AKI after reperfusion. The activated inflammatory response was seen in the liver tissue as well as renal tissue after reperfusion [37]. The graft ischemia/reperfusion injury induced the infiltration of leukocytes and liver cell injury. Proinflammatory cytokines released from the reperfused liver graft can act on the kidney tissue. IL-6 and TNF-a will trigger leukocyte–endothelium interactions and microcirculatory dysfunction, and alter renal microvascular O_2_ distribution and promote organ damage [25,38]. In addition, the venous warm ischemia from suprarenal IVC occlusion can also activate inflammatory response in renal tissue and aggravate kidney injury [39]. Macrophage activation and neutrophilic infiltration appear to be exaggerated during venous occlusion and increase severity of renal injury [39]. Thus, the renal microcirculation might be finally affected after reperfusion and estimation of renal perfusion is significantly limited [40]. The finding that no correlation between the RI values and the sCr and Cys and NGAL levels after reperfusion might suggested that Doppler ultrasound of renal arteries is not helpful in diagnosing renal function impairment post-LT.

Our study has several limitations. We focused on the short-term changes of renal RI and renal function after reperfusion, some important factors related to RI and renal function in human LT are not included in our study. We did not use inotropic agents, vasoconstrictors, immunosuppressive anti-rejection drugs. Second, we did not measure the FE_Na_ during the anhepatic phase because of the insufficient urinary volume for analysis. In addition, the operation was carried out between the rats with normal preoperative liver function, some patients with chronic liver failure have hepatorenal syndrome, increased RI and impaired renal function before the operation.

## Conclusion

In conclusion, there were major changes in renal RI during operation in liver transplantation rat model. RI increased significantly in the anhepatic stage but decreased drastically after early reperfusion of graft compared with baseline. Intrarenal RI was unable to assess renal function in a rat liver transplantation model.

## Competing interests

The authors declare that they have no competing interests.

## Authors’ contributions

KHY participated in the design of the study, performed the statistical analysis and drafted the manuscript. CF performed the Doppler examinations. HY carried out the liver transplantation rat model. WLJ carried out the immunoassays. WLQ collected data and analyzed data. ZSM contributed substantially to the writing of the manuscript. ZSS conceived of the study, and participated in its design and coordination and helped to draft the manuscript. All authors read and approved the final manuscript.

## Pre-publication history

The pre-publication history for this paper can be accessed here:

http://www.biomedcentral.com/1471-2369/14/55/prepub
